# Prognostic significance of the neutrophil-to-lymphocyte ratio with distal cholangiocarcinoma patients

**DOI:** 10.1097/MD.0000000000022827

**Published:** 2020-10-23

**Authors:** Fengming Ji, Qiang Kang, Lianmin Wang, Lixin Liu, Yang Ke, Ya Zhu, Naiqiang Zhang, Shifeng Xiong, Yuehua Li, Hao Zou

**Affiliations:** a Department of Hepatobiliary Surgery, The Second Affiliated Hospital; b Urology Department of The Affiliated Children's Hospital of Kunminng Medical University, Kunming Chlidren's Hospital, Key Laboratory of Children's Major Disease Research, Kunming Medical University; c Department of General Surgery, Kunming Traditional Chinese Medicine Hospital, Kunming, Yunnan, PR China.

**Keywords:** distal cholangiocarcinoma, neutrophil-to-lymphocyte ratio, independent risk factor, overall survival, prognosis

## Abstract

**Background:**

The present study aimed to investigate the prognostic value of the neutrophil-to-lymphocyte ratio (NLR) in distal cholangiocarcinoma (DCC) following radical surgery.

**Methods:**

The clinicopathological data of 59 patients with DCC were retrospectively reviewed. Patients were treated by radical surgery and diagnosed by postoperative pathology at the Second Affiliated Hospital of Kunming Medical University (Yunnan, China), between July 2015 and December 2017. The optimal cut-off value for the NLR was determined by generating receiver operating characteristic (ROC) curves. Kaplan–Meier survival analysis and Cox proportional hazards models were used to determine the risk factors and independent risk factors influencing the prognosis of patients with DCC.

**Results:**

According to the ROC curve, the optimal cut-off value for the NLR was 2.933. The results of Kaplan–Meier survival analysis and the Cox proportional hazards model showed that carbohydrate antigen 125, NLR, perineural, vascular and fat invasion, regional lymph node metastasis, and the American Joint Committee on Cancer stage were risk factors for DCC; the only independent risk factor to affect the prognosis of DCC patients was the NLR.

**Conclusions:**

The preoperative NLR plays an important guiding role in evaluating the prognosis of patients with DCC, and an increase in the NLR is associated with poor patient prognosis.

## 1 Introduction

Cholangiocarcinoma is a malignant tumor originating from the biliary epithelium. It is a frequently fatal malignancy associated with poor patient prognosis, and accounts for∼3% of all gastrointestinal tumors. Based on anatomical location, cholangiocarcinoma is broadly categorized as intrahepatic or extrahepatic cholangiocarcinoma. The World Health Organization has further subdivided extrahepatic cholangiocarcinoma into hilar cholangiocarcinoma (HCC) and distal cholangiocarcinoma (DCC), which are separated by their location in the cystic duct *outside* of the liver (at the confluence of the right and left ducts for HCC, and at the point closest to the intestine for DCC). At present, ∼60% to 70% of extrahepatic cholangiocarcinomas are hilar in nature, and DCC accounts for the remaining 30% to 40%.^[1]^ Currently, the only curative treatment for DCC is surgical resection. However, as the early symptoms are not obvious, the majority of patients miss the opportunity to undergo surgery, and only one third are suitable for radical resectionat the time of diagnosis. Furthermore, the median survival time after radical surgery is reported to be only 24 mouths, with a 5-year survival rate of <10%.^[2]^ The poor prognosis after surgery is primarily due to tumor recurrence and metastasis; therefore, it is necessary to identify effective prognostic indicators to predict postoperative survival in patients with DCC, thus guiding the selection of treatment methods to improve patient prognosis.

Lymph node metastasis, perineural and vascular invasion, and tumor size are among the factors closely associated the progression and prognosis of malignant tumors.^[3,4]^ However, patient prognosis is not only associated with the biological characteristics of the tumor, but also the physical condition of the host. An increasing number of studies have demonstrated that the inflammatory response plays a key role in the changes to the microenvironment of normal tissue, and thus is an important factor for tumor occurrence and development.^[5,6]^ Several biomarkers have been identified as prognostic risk factors for various types of cancer, including interleukin-6 (IL-6) in ovarian cancer,^[7]^ C-reactive protein (CRP) in hepatocellular carcinoma,^[8]^ CRP and albumin (ALB) ratio in cervical cancer,^[9]^ aspartate aminotransferase (AST) and the neutrophil ratio in intrahepatic cholangiocarcinoma,^[10]^ and the platelet-to-lymphocyte ratio (PLR) in breast cancer.^[11]^ The present study aimed to investigate the potential correlation between the NLR and the prognosis of DCC patients after radical surgery.

## 2 Methods

### 2.1 Patients and analytical factors

A retrospective study was conducted using a database of 59 DCC patients who underwent radical resection at the Second Affiliated Hospital of Kunming Medical University (Yunnan, China), between July 2015 and December 2017; 33 men and 26 women were included, with a mean age of 57.5 years (range, 37–76 years). Factors analyzed included sex, age, ALB, alanine transaminase (ALT), AST, total bilirubin (TB), direct bilirubin (DB), NLR, PLR, carcinoembryonic antigen (CEA), carbohydrate antigen 125 (CA125), carbohydrate antigen 19-9 (CA19-9), Ki 67, perineural invasion, vascular invasion, fat invasion, total lymph node metastasis, regional lymph node metastasis, and American Joint Committee on cancer (AJCC) stage. The cut-off values for ALB (35 g/L), ALT (40 U/L,) AST (40 U/L), CEA (5 ng/L), CA125 (35 U/ml) and CA19-9 (35 U/ml) according to the normal biological levels. The cut-off value for Ki 67 was set as 20% according to the research of Rijken et al,^[12]^ and the AJCC stage was classified using the guidelines outlined in the eighth edition of the AJCC staging manual.

### 2.2 Inclusion and exclusion criteria

All hematological examination data were obtained before admission, prior to the administration of antibiotics, hepatoprotective drugs, or preoperative drainage for obstructive jaundice. The inclusion criteria included:

1. Complete clinical information; and
2. that patients underwent radical surgery and were diagnosed with DCC by postoperative pathology.

The exclusion criteria included:

1. Incomplete clinical data;
2. patients undergoing palliative surgery only;
3. patients with a pre- or postoperative history of radiotherapy and chemotherapy; and
4. patients diagnosed with ampulla carcinoma.

### 2.3 Follow-up

According to the inclusion and exclusion criteria, a total of 59 patients were included in this study. All patients were followed up by telephone or outpatient appointment, and the follow-up deadline was July 1, 2018.

### 2.4 Methods

The research database was established using the 2010 version of Office Excel, and statistical analyses were performed using SPSS version 19.0. Kaplan–Meier analysis was used to determine the risk factors for DCC, and those identified by univariate analysis were also subjected to multivariate analysis. Hazard ratios and 95% confidence intervals (95% CI) were estimated using forward stepwise Cox proportional hazard model analysis. Receiver operating characteristic (ROC) curve analysis was performed to determine the optimal cut–off values for the NLR, PLR, T, and DB. The patients were then assigned to high or low NLR groups according to the NLR cut-off value Categorical variables between the different NLR groups were compared using Pearsons Chi-Squared test, and *P* < .05 was considered to indicate a statistically significant difference.

## 3 Results

### 3.1 General patient data and oncological characteristics

The data of 33 male (55.93%) and 26 female (44.07%) patients were used in the present study. The mean patient age was 57.5 years (range, 37–76 years). During the follow-up period, a total of 22 patients died; the mean postoperative time and median survival time of the 59 patients were 11.92 and 12 months, respectively, and the 1- and 3-year survival rates were 63.1% and 50.9%, respectively. Detection of preoperative serum tumor markers revealed an increase in CEA in 52 patients, 51 patients with increased CA125 and 15 with increased CA19-9. In the postoperative pathological examination, 27 patients exhibited Ki67 > 20%, perineural invasion occurred in 30 patients, vascular invasion in 14 patients, fat invasion in 17 patients, total lymph node metastasis in 20 patients and regional lymph node metastasis in 16 patients. According to the eighth edition of the AJCC DCC staging system, 50 were I/II stage and 9 were III/IV stage patients.

### 3.2 ROC curve analysis

**Figure 1:**
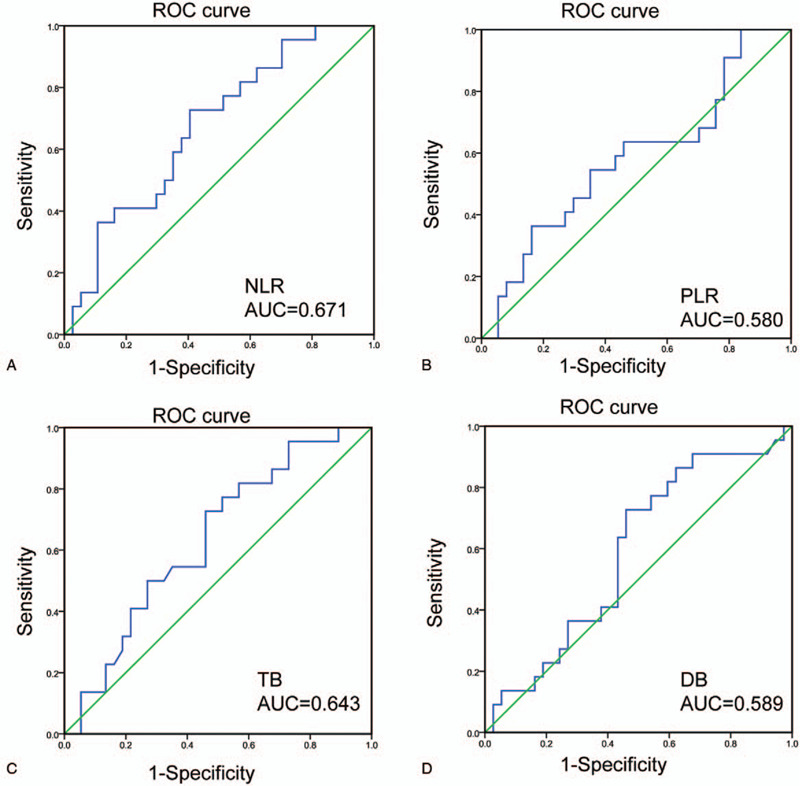
ROC analysis was performed to determine the optimal cut-off value of NLR, PLR, TB, DB in patients with DCC after radical surgery.

The NLR was calculated using the following formula: NLR = neutrophil count/lymphocyte count. According to the ROC curve analysis results, the optimal cut-off value for the NLR was 2.933 when predicting postoperative prognosis. The area under the ROC curve for survival status was 0.671, with a 95% CI of 0.532 to 0.810 (*P* = .029) (Fig. 1). Subsequently, all patients were divided into high (NLR > 2.933, n = 31) and low (NLR ≤2.933, n = 28) NLR groups. Using the same method, the optimal cut-off values for PLR, TB and DB were determined to be 269.921, 175.5, and 126.4, respectively. The ROC curves are displayed in Fig. 1.

### 3.3 Relationship between the NLR and clinicopathological characteristics in DCC

Pearsons Chi-Squared test was used to determine potential statistical differences in the distribution of categorical variables between the NLR groups. The results indicate that sex (*P* = .000), AST (*P* = .019) and PLR (*P* = .035) were statistically different between the two NLR groups (Table 1).

**Table 1:**
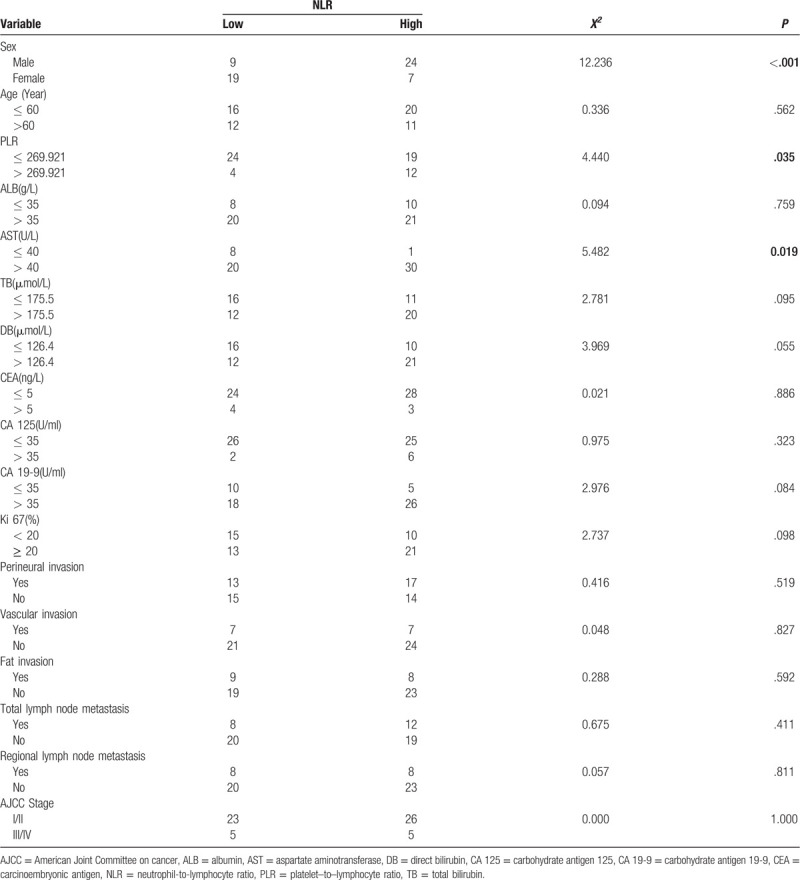
Relationship between neutrophil-to-lymphocyte ratio and clinicopathological characteristics in 59 distal cholangiocarcinoma patients.

### 3.4 Univariate and multivariate survival analyses

**Figure 2:**
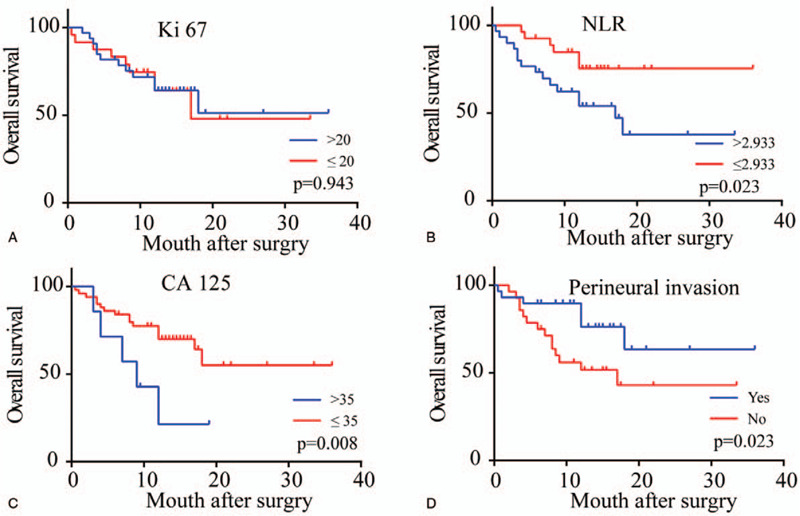
Kaplan–Meier survival curves of patients with DCC after radical surgery by Ki 67 (A), NLR (B), CA 125 (C), perineural invasion (D). *P* values were obtained by log-rank tests.

**Figure 3:**
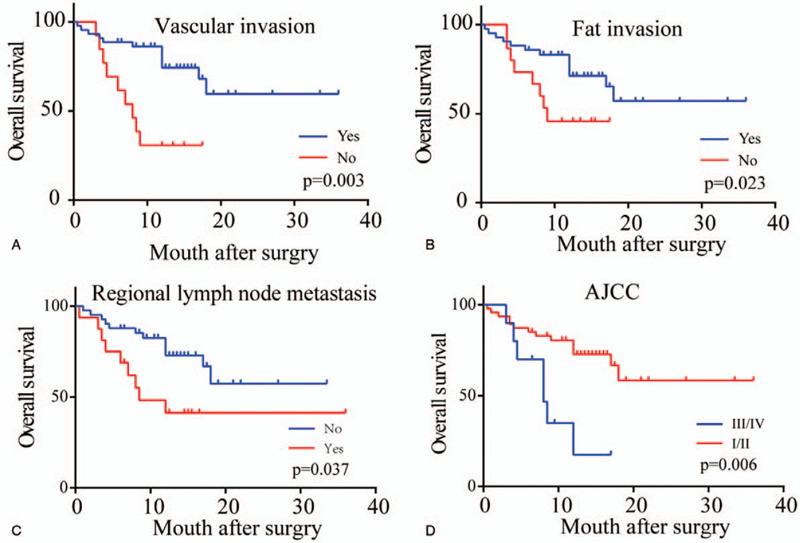
Kaplan–Meier survival curves of patients with DCC after radical surgery by vascular invasion (A), fat invasion (B), regional lymph node metastasis (C), AJCC stage (D). *P* values were obtained by log-rank tests.

Univariate analysis revealed that the NLR (*P* = .023), CA125 (*P* = .008), perineural invasion (*P* = .023), vascular invasion (*P* = .003), fat invasion (*P* = .023), regional lymph node metastasis (*P* = .037) and AJCC stage (*P* = .006) were all risk factors for the overall survival of DCC patients following radical surgery (Figs. 2 and 3, Table 2). Multivariate analysis revealed that only the NLR (*P* = .024) was an independent risk factor for the prognosis of patients with DCC (Table 3).

**Table 2:**
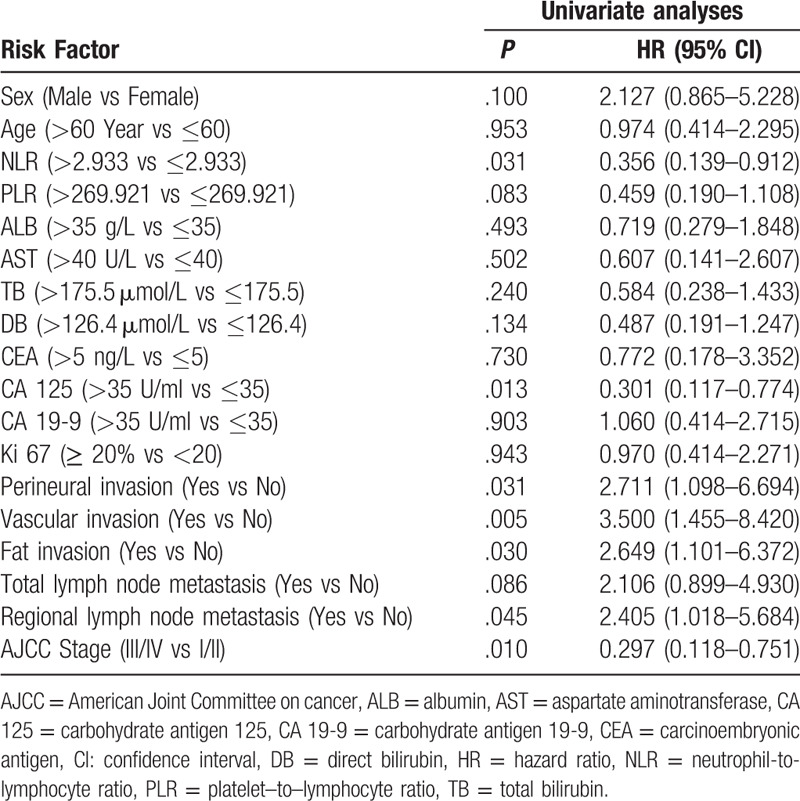
Univariate analysis of factors associated with overall survival in 59 distal cholangiocarcinoma patients.

**Table 3:**
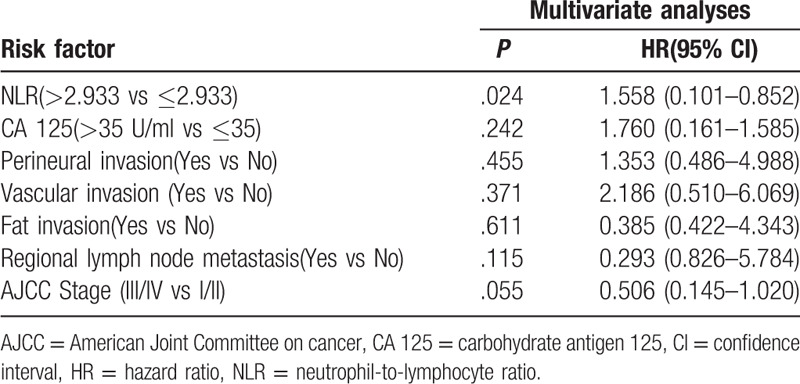
Multivariate analysis of factors associated with overall survival in 59 distal cholangiocarcinoma patients.

## 4 Discussion

An increasing number of studies has indicated that a high preoperative NLR is associated with poor patient outcome in various types of cancer, including pancreatic cancer, renal cancer, non-small cell lung cancer, gastric cancer, hepatocellular carcinoma, and various other solid tumors.^[6,13–16]^ Using univariate and multivariate analysis, the present study investigated the relationship between multiple clinicopathological factors and the prognosis of DCC patients. It was ultimately confirmed that a high preoperative NLR (>2.933) is an independent risk factor for the overall survival rate of patients with DCC. Although numerous studies have determined that regional lymph node metastasis, perineural invasion, vascular invasion, and tumor differentiation have prognostic value in predicting the progression of malignant tumors, these indicators can only be assessed following radical surgery.^[17]^ The NLR has the advantages of being economical and conveniently accessible, but can also be obtained preoperatively, thus has great practical value in a clinical setting.

As one of the 10 characteristics of malignant tumors, the inflammatory response has attracted a great deal of attention. In 1863, Rudolf Virchow et al^[18]^ identified a large degree of inflammatory cell infiltration in tumor tissues, and first proposed that chronic inflammation was the origin of malignant tumors. A large number of studies have confirmed that the inflammatory response serves an important role in the occurrence, development and metastasis (as well as other stages) of tumorigenesis. Inflammation can promote tumorigenesis through gene mutation, genomic instability, and epigenetic modification. It can also activate the tissue repair response and induce the proliferation of precancerous cells.^[19]^ Inflammation also stimulates angiogenesis, leads to immunosuppression, and promotes the formation of tumor microenvironments that ultimately result in metastasis.^[20]^

Neutrophils are white blood cells that play important roles in the inflammatory response. They have strong antibacterial phagocytic functions and are an important barrier against exogenous infection.^[21]^ Neutrophils play opposing roles in the process of tumorigenesis and development. On the one hand, neutrophils destroy tumor cells by directly releasing antibacterial and cytotoxic substances, or immune mediators that activate the relevant anti-tumor cells. On the other hand, neutrophils also release cytotoxic substances that cause DNA damage in epithelial cells and activate oncogenes.^[22]^ Neutrophil infiltration into precancerous lesions is mediated by chemokine receptor 2 ligands. Once activated, precancerous tissue neutrophils release reactive oxygen and nitrogen species, and proteases, which collectively promote tissue carcinogenesis. Nitric oxide synthase, arginase 1 and matrix metalloproteinase-9 released by neutrophils activate the immune escape mechanism whilst inhibiting the anti-tumor properties of CD8+ T lymphocytes, which ultimately results in tumor metastasis.^[23,24]^

Lymphocytes are an important immune barrier against tumor cells. Karin et al^[25]^ established a tumor-susceptible mouse model lacking both B and T lymphocytes. They demonstrated that B lymphocytes were necessary for establishing chronic inflammation, and that activated B lymphocytes may regulate cancer development by altering the levels of circulating cytokines and/or chemokines. T lymphocytes not only destroy tumor cells directly, but can also induce apoptosis of target cells by Fas-FasL binding on the surface of tumor cells. Furthermore, Tumor-infiltrating CD4+ T lymphocytes induce apoptosis by activating CD8+ T lymphocytes and releasing cytotoxic factors.^[26]^

Neutrophils can inhibit adaptive immune responses and reduce the burden on activated lymphocytes.^[27]^ Therefore, a high NLR may indicate an increase in neutrophil-dependent inflammatory responses, and a corresponding decrease in the lymphocyte-mediated antitumor immune response. Neutrophil-dependent responses can promote tumor invasiveness and thus a poor patient prognosis.^[6]^ Therefore, the NLR is an important indicator of the balance between the inflammatory and immune responses.

At present, the applications of the NLR are not limited to the prognostic evaluation of malignant tumor patients. Patients with laryngeal squamous cell carcinoma who present with a preoperative NLR > 2.5 showed a higher risk of developing pharyngocutaneous fistula in the postoperative setting of total laryngectomy (*P* = .007).^[28]^ A study of Julia et al^[29]^ demonstrated that the NLR has important clinical significance for the identification of sepsis and bacteraemia in patients with burn injuries (*P* = .000). Furthermore, Jeong-ju et al^[30]^ highlighted that a high NLR is an independent risk factor for poor prognosis in patients with primary cholangitis (*P* < .01), and Lou et al^[31]^ found that an NLR > 11 had important predictive value for the weaning failure of ICU patients (*P* < .001). Zhou et al^[32]^ has revealed that NLR > 6.5 often indicates the occurrence of a strangulated inguinal hernia in patients with inguinal hernia (*P* < .001), and Ali et al^[33]^ suggest that a preoperative NLR > 5 is an independent predictive marker of 30 day morbidity in ruptured abdominal aortic aneurysms (*P* = .02).

In the present study, a systematic review of the preoperative hematological and pathological indices of 59 DCC patients revealed that the preoperative NLR was an independent risk factor affecting the overall survival rates of DCC patients. However, the study had several limitations. Firstly, bias when selecting cases was inevitable in a single-center retrospective study. Secondly, due to objective factors, procalcitonin, CRP, IL-6, and other important inflammatory indicators were not assessedin this study. Therefore, further prospective studies are required to confirm the results of the present study.

## 5 Conclusions

The results of the present study reveal that a preoperative NLR > 2.933 is an independent risk factor for poor prognosis in patients with DCC. As an economical and convenient preoperative indicator, the NLR can be used to preliminarily estimate the prognosis of patients, and to formulate adjuvant treatment and follow-up plans. Many studies have indicated that a high NLR is associated with the poor prognosis of malignant tumor patients; however, whether clinical intervention to reduce the preoperative NLR has clinical significance in improving long-term prognosis remains to be elucidated.

## Author contributions

**Data curation:** Lianmin Wang.

**Formal analysis:** Fengming Ji, Lixin Liu.

**Funding acquisition:** Hao Zou.

**Methodology:** Qiang Kang, Yang Ke, Ya Zhu.

**Project administration:** Hao Zou.

**Software:** Shifeng Xiong.

**Visualization:** Naiqiang Zhang.

**Writing – original draft:** Fengming Ji, Qiang Kang, Lianmin Wang.

**Writing – review & editing:** Yuehua Li, Hao Zou.
